# A Real-World Study of the Effectiveness and Safety of Semaglutide for Weight Loss

**DOI:** 10.7759/cureus.59558

**Published:** 2024-05-02

**Authors:** Ploutarchos Tzoulis, Michael Batavanis, Stephanie Baldeweg

**Affiliations:** 1 Department of Metabolism and Experimental Therapeutics, University College London, London, GBR; 2 School of Clinical Medicine, University of Cambridge, Cambridge, GBR; 3 Center for Obesity and Metabolism, Department of Experimental and Translational Medicine, University College London, London, GBR

**Keywords:** glp-1, weight loss, overweight, semaglutide, obesity

## Abstract

Introduction

Recent randomized controlled trials (RCTs) have shown the great efficacy of semaglutide in achieving significant weight loss in overweight and obese adults. However, real-world data about its effectiveness are still limited. This study evaluated the effectiveness and adverse events of semaglutide for weight management in a real-life setting, excluding patients with diabetes mellitus (DM).

Methods

This is a retrospective chart review of 40 overweight or obese individuals with a median age of 47 years, weight of 111.7 kg, and body mass index (BMI) of 39.7 kg/m^2 ^who were prescribed semaglutide for weight management.

Results

After three months of semaglutide administration, the median weight reduction was 7.4 kg (6.6% of the baseline weight), with 28 (70%) and eight patients (20%) achieving greater than 5% (5.6 kg) and 10% (11.2 kg) weight loss, respectively. Among 25 patients with six-month data, 22 (88%), 17 (68%), and eight (32%) patients exceeded 5% (5.6 kg), 10% (11.2 kg), and 15% (16.8 kg) weight loss, respectively. The maintenance semaglutide dose was 1 mg in 16 cases and 2 mg in nine cases, leading to a similar weight loss of 13.6% (14.9 kg) and 12.8% (14 kg), respectively. Relatively low response rates were observed in males, with seven responders out of 12 (58.4%) compared to 24 out of 28 (85.8%) in females (P value = 0.057), and in five out of nine (55.6%) among those with a history of psychiatric disease. The rate of adverse events was 26 out of 40 patients (65%), mostly mild to moderate and of short duration, leading to discontinuation in only a single case (2.5%).

Conclusion

This retrospective study demonstrated the significant effectiveness of semaglutide for weight loss, even at lower than approved maintenance doses, combined with a good safety profile. Therefore, semaglutide may dramatically change the landscape of obesity treatment.

## Introduction

Obesity is nowadays recognized as a multifactorial, chronic, relapsing, progressive disease. It is characterized by the presence of excessive adipose tissue, most commonly defined as a body mass index (BMI) greater than or equal to 30 kg/m^2^, calculated as weight in kilograms divided by height in meters squared (kg/m^2^) [1]. Obesity is associated with an increased risk for several diseases, such as type 2 diabetes mellitus (DM), dyslipidemia, hypertension, cardiovascular disease, several types of cancer, hepatobiliary disease, obstructive sleep apnea, and osteoarthritis, and with excess mortality. Over the last three decades, there has been an exponential increase in rates of obesity, which has become a global epidemic and a major public health problem [2].

People with obesity should receive personalized care from a multidisciplinary team, including a combination of medical nutrition therapy and physical activity, supplemented by psychological and behavioral interventions. In most cases, lifestyle interventions have limited efficacy and durability with the long-term maintenance of weight loss remaining a big challenge [3]. Despite this unmet clinical need, anti-obesity medications (AOMs) have been underutilized in routine clinical practice, with less than 1.5% of those eligible for pharmacotherapy having received AOMs [4]. Until 2021, three AOMs were available in Europe, namely, orlistat (a lipase inhibitor), a combination of naltrexone and bupropion (an opioid receptor antagonist combined with a dopamine/norepinephrine reuptake inhibitor), and liraglutide, a daily injectable glucagon-like peptide-1 (GLP-1) agonist. These AOMs result in a mean one-year placebo-subtracted weight loss of less than 10% [5], while greater than 10% weight loss is required to improve common weight-related comorbidities and complications, such as obstructive sleep apnea and metabolic dysfunction-associated liver disease [5,6].

In 2021, semaglutide was approved by both the US Food and Drug Administration (FDA) and the European Medicines Agency (EMA) for chronic weight management in patients with either a BMI greater than 30 kg/m^2^ or a BMI greater than 27 kg/m^2^ and at least one weight-related condition. This indication followed its initial approval for the treatment of type 2 diabetes mellitus (DM) in 2017. Semaglutide, a weekly injectable GLP-1 agonist, reduces appetite and increases satiety through the activation of GLP-1 receptors in the hypothalamus and the hindbrain, leading to reduced energy intake. A global program of phase 3 clinical trials evaluating the effect of semaglutide use for weight management in people with obesity, the Semaglutide Treatment Effect in People with Obesity (STEP) trials, has reported an average placebo-subtracted weight loss percentage of 12.5%, with more than half of the participants achieving 15% or more weight loss [5,7]. Therefore, semaglutide has become the first AOM that results in a greater than 10% average weight loss over that attributable to lifestyle interventions [5], generating widespread public interest and leading to global supply shortages. Besides semaglutide, numerous studies are currently exploring the potential synergistic effect of targeting multiple gut peptides. Novel agents, such as tirzepatide, a dual GLP-1 and glucose-dependent insulinotropic polypeptide (GIP) agonist; the combination of semaglutide with cagrilintide, an amylin analogue; and retatrutide, a triple agonist of GLP-1, GIP, and glucagon receptors have produced bodyweight reduction in the range of 17%-24%, surpassing that achieved with semaglutide monotherapy. In addition, oral formulations of GLP-1 agonists, such as once-daily oral semaglutide and orforglipron, have led to similar weight loss with that of injectable semaglutide. These promising results suggest that a variety of novel pharmacotherapies will become available in the next few years, revolutionizing the treatment of obesity [2].

A series of randomized controlled trials (RCTs) have consistently demonstrated the great efficacy of semaglutide for weight management [7-12]. Despite the wealth of high-quality evidence, there is a paucity of real-life data limited to one cohort study [13]. The aim of this real-world retrospective study was to evaluate the effectiveness and adverse events of semaglutide therapy for weight management in individuals with obesity in day-to-day clinical practice.

## Materials and methods

Study design

This is a retrospective review of the medical records of all individuals treated with semaglutide for weight management in an endocrine clinic in Athens, Greece. Individuals were started on semaglutide in the time period between November 2021 and November 2022, with no more patients initiated on semaglutide afterward due to its supply shortages. This retrospective review of patients’ medical charts was conducted according to Good Clinical Practice guidelines and the Declaration of Helsinki. It was granted an exemption from requiring ethical approval since it was a clinical audit designed to capture the clinical outcomes of patients commenced on semaglutide within its licensed use for weight loss and all the procedures being performed were part of the routine care.

Patient selection

This retrospective observational study included all adults who met three criteria: (i) having a BMI greater than 30 kg/m^2^ regardless of comorbidities or, alternatively, a BMI greater than 27 kg/m^2^ combined with, at least, one weight-related complication; (ii) being prescribed semaglutide for weight management; and (iii) having a minimum of three-month duration of semaglutide therapy with data recorded at the end of this period. At the baseline, all individuals underwent screening for prediabetes and diabetes, with the diagnosis of DM being an exclusion criterion. Individuals who had undergone bariatric surgery or had taken other AOMs in the past were not excluded, while the concomitant administration of other AOMs during the study period was an exclusion criterion.

Procedures

Semaglutide use required patient self-funding and was commenced at a dose of 0.25 mg once weekly for the first four weeks. Thereafter, according to the standard titration regimen [7], the dose was increased after four weeks to 0.5 mg and after eight weeks to 1 mg. After the completion of a 12-week semaglutide therapy, a decision for maintenance semaglutide dose was made on a case-by-case basis, taking into account clinical response, as well as drug affordability. All individuals received counselling sessions about nutrition and regular exercise at the time of semaglutide initiation and every 12 weeks thereafter by an endocrinologist, following the principles of motivational interviewing. It was left to the discretion of each individual whether they sought regular dietician input, participated in a structured physical activity program, or received behavioral/psychological therapy.

Data collection

For each individual, data were recorded at the baseline and in three-month intervals following semaglutide initiation about demographic parameters, anthropometric characteristics, and a wide range of laboratory parameters. Information was also collected about the presence of various comorbidities according to past medical history, medication use, and available laboratory and imaging tests. They included a total of 12 weight-related comorbidities, such as prediabetes, dyslipidemia, metabolic dysfunction-associated fatty liver disease (MAFLD), weight-related reproductive disorders, obstructive sleep apnea, psychiatric diseases, hypertension, gastroesophageal reflux disease, knee osteoarthritis, coronary artery disease, cancer, and airway disease. In addition, information on any adverse events was collected at each visit. Weight was measured on the same calibrated scale during each office visit. According to their BMI, individuals were classified into four subgroups: (i) BMI of 27.0-29.9 kg/m^2^, overweight; (ii) BMI of 30-34.9 kg/m^2^, class I obesity; (iii) BMI of 35-39.9 kg/m^2^, class II obesity; and (iv) BMI of ≥40 kg/m^2^, class III obesity.

End points and statistical analysis

The primary end point was the percentage weight loss at three and six months after semaglutide initiation. The secondary end points included (i) the percentage of patients who achieved more than 5%, more than 10%, and more than 15% weight loss at each time point; (ii) the change in markers of glucose metabolism; and (iii) the frequency and severity of adverse events. The subgroup analysis for the gender and BMI category was conducted in order to examine the impact of baseline characteristics on the magnitude of semaglutide-induced weight loss. The Statistical Package for Social Sciences (SPSS) software (version 22.0) (IBM SPSS Statistics, Armonk, NY) was used for all statistical analyses. Data were summarized using descriptive statistics, with continuous variables being expressed as median and interquartile range (IQR) and categorical variables as percentages. The association of three-month percentage weight loss with gender and baseline BMI was assessed with the Mann-Whitney U test and the Pearson correlation coefficient, respectively. A chi-squared test was employed to assess the gender distribution of non-responders to semaglutide who were defined as individuals who achieved neither a three-month weight loss of 3% nor a six-month weight loss of 5%. A P value of <0.05 was chosen to denote statistical significance.

## Results

Study participants

Semaglutide for weight management was prescribed for a total of 43 patients who were overweight or obese, but the analysis included 40 individuals (28 females and 12 males) following the exclusion of three individuals owing to the lack of three-month data. Prior to semaglutide initiation, all patients underwent the measurement of fasting plasma glucose, which was often supplemented by glycated hemoglobin (HbA1c) and, in selected cases, by an oral glucose tolerance test. According to the American Diabetes Association criteria, the results were interpreted, and individuals were classified into the following groups: normoglycemia, prediabetes, and DM [14]. Out of 40 patients, five individuals had undergone bariatric surgery in the past, including gastric banding in four cases and sleeve gastrectomy in one case. Prior to semaglutide treatment, four patients had received AOMs in the past, namely, a combination of naltrexone and bupropion in three cases and liraglutide in one case. Six-month data were available in 25 out of 40 (62.5%) semaglutide-treated patients, with the remaining 15 individuals having not completed six-month treatment due to a variety of reasons, including severe side effects in one case, poor three-month response in four cases, and supply shortages/drug affordability in 10 cases.

Baseline characteristics

The demographic and clinical baseline characteristics are summarized in Table 1. All patients were of White Greek ethnic origin, mostly females (28/40 or 70%), with a median age of 47, ranging from 23 to 65 years old. Among the 40 individuals, the median weight was 111.7 kg, and BMI was 39.7 kg/m^2^, with half of the patients having class III obesity. With regard to glucose metabolism, 20 individuals had impaired fasting glucose (100-125 mg/dL), and 15 patients had glycated hemoglobin (5.7%-6.4%), indicating an overall prevalence for prediabetes as per the American Diabetes Association criteria of 57.5% (23 out of 40). Fasting insulin values were available in 28 participants, with insulin resistance, defined as high homeostatic model assessment for insulin resistance (HOMA-IR) ≥ 1.9 [15], being reported in 24 out of 28 cases (86%), while severe insulin resistance, defined as HOMA-IR exceeding 4, being recorded in 15 out of 28 participants (53.5%). Dyslipidemia was present in 29 out of 40 (72.5%) patients, classified into mixed dyslipidemia in 13 cases (32.5%), isolated hypercholesterolemia in 13 cases (32.5%), and isolated hypertriglyceridemia in three cases (7.5%), while low serum high-density lipoprotein (HDL) concentration (less than 50 mg/dL in females and less than 40 mg/dL in males) was reported in 16 individuals (40%). In total, 17 out of 40 patients (42.5%) combined the presence of at least three criteria, which constituted the diagnosis of metabolic syndrome according to the National Cholesterol Education Program (NCEP) Adult Treatment Plan III (ATP III) definition [16]. Other common comorbidities were MAFLD, reproductive abnormalities, psychiatric disorders, and obstructive sleep apnea, with 32 out of 40 individuals (80%) having two or more weight-related medical conditions.

**Table 1:** Baseline demographic and clinical characteristics *The marked characteristics are expressed as median (interquartile range, IQR) ^1^HOMA-IR was calculated with the following formula: fasting glucose (mg/dL) multiplied by fasting insulin (mIU/L) and divided by 405 ^2^Prediabetes was defined as fasting plasma glucose of 100-125 mg/dL or HbA1c of 5.7%-6.4% or two-hour plasma glucose of 140-199 mg/dL during oral glucose tolerance test with a glucose load containing 75 g of glucose, in line with the most recent American Diabetes Association criteria ^3^Dyslipidemia was defined as meeting at least one of the following three criteria: serum total cholesterol > 200 mg/dL or LDL cholesterol > 130 mg/dL or triglycerides > 150 mg/dL, according to the National Cholesterol Education Program (NCEP) Adult Treatment Plan III (ATP III) ^4^Hypercholesterolemia was defined as serum total cholesterol > 200 mg/dL ^5^Hypertriglyceridemia was defined as serum triglycerides > 150 mg/dL ^6^Low HDL serum concentration was defined as < 40 mg/dL in males and < 50 mg/dL in females ^7^Reproductive comorbidities included a diagnosis of polycystic ovarian syndrome (PCOS) in females and hypogonadotropic hypogonadism in males ^8^Psychiatric weight-related conditions included depression, generalized anxiety disorder, and bipolar disorder or schizophrenia ^9^Hypertension was defined as receiving active treatment or blood pressure exceeding 130/85 mmHg ^10^Cancer was defined as an active or past medical history of cancer of any origin ^11^Airway disease included asthma and chronic obstructive pulmonary disease (COPD) BMI, body mass index; HOMA-IR, homeostatic model assessment for insulin resistance; HDL, high-density lipoprotein; LDL, low-density lipoprotein; MAFLD, metabolic dysfunction-associated fatty liver disease

Characteristic	Patients (N = 40)
Age, years*	47 (13)
Age ranges, number (%)	
<40	6 (15%)
40-49	17 (42.5%)
50-59	12 (30%)
≥60	5 (12.5%)
Gender, number (%)	
Female	28 (70%)
Male	12 (30%)
Ethnic origin, number (%)	
White Greek	40 (100%)
Smoking status, number (%)	
Never smoked	20 (50%)
Currently smoking	16 (40%)
Ex-smokers	4 (10%)
Body weight, kg*	111.7 (33.8)
BMI, kg/m^2^*	39.7 (9.2)
BMI categories, number (%)	
Overweight	3 (7.5%)
Class I obesity	10 (25%)
Class II obesity	7 (17.5%)
Class III obesity	20 (50%)
Markers of glucose metabolism	
Glycated hemoglobin (HbA1c), %*	5.5 (0.7)
Fasting glucose, mg/dL*	101 (18)
Fasting insulin, mIU/L*	17 (18)
^ 1^HOMA-IR index*	4.6 (4.4)
^ 2^Prediabetes, number (%)	23 (57.5%)
Lipid profile	
Total cholesterol, mg/dL*	202 (33)
LDL cholesterol, mg/dL*	120 (40)
HDL cholesterol, mg/dL*	49 (24)
Total triglycerides, mg/dL*	119 (111)
^ 3^Dyslipidemia, number (%)	29 (72.5%)
^ 4^Hypercholesterolemia, number (%)	26 (65%)
^ 5^Hypertriglyceridemia, number (%)	15 (37.5%)
^ 6^Low HDL cholesterol, number (%)	16 (40%)
Comorbidities, number (%)	
MAFLD	18 (45%)
^ 7^Reproductive abnormalities	11 (27.5%)
Obstructive sleep apnea	9 (22.5%)
^ 8^Psychiatric diseases	9 (22.5%)
^ 9^Hypertension	7 (17.5%)
Gastroesophageal reflux disease	6 (15%)
Knee osteoarthritis	4 (10%)
Coronary heart disease	2 (5%)
^ 10^Cancer	2 (5%)
^ 11^Airways disease	1 (2.5%)
Number of comorbidities, number (%)	
None	2 (5%)
1	8 (20%)
2	9 (22.5%)
3	7 (17.5%)
4	6 (15%)
≥5	8 (20%)

Changes in body weight

**Figure 1:**
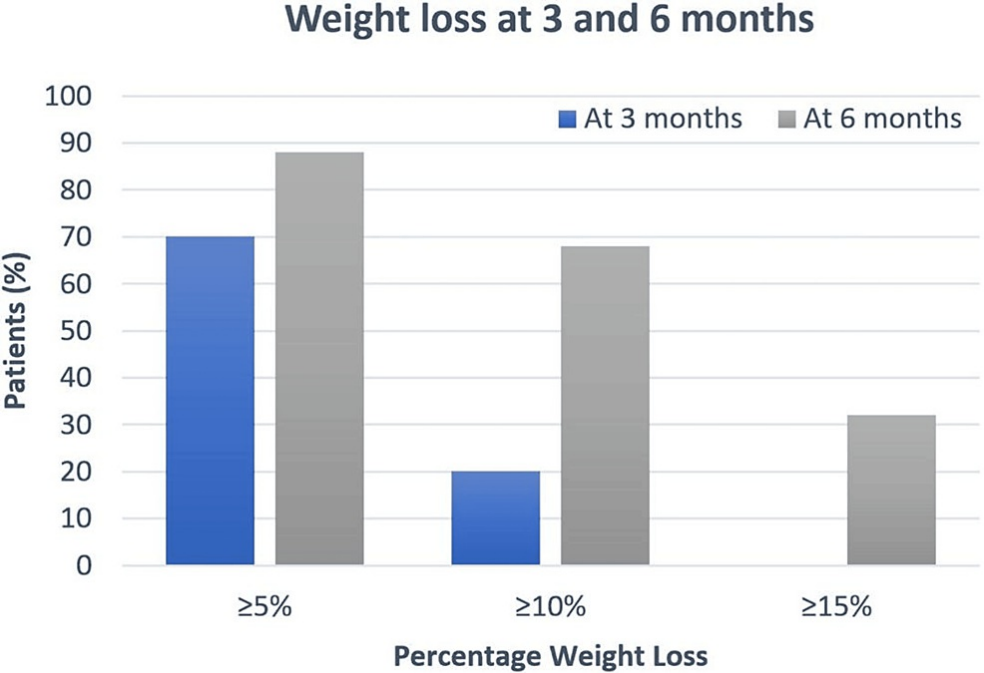
Percentage of patients who achieved ≥5%, ≥10%, and ≥15% weight loss at three and six months The blue bars denote three-month data. The grey bars denote six-month data

After three months of semaglutide administration, the median (IQR) weight loss was 7 (5.3) kg, equivalent to 6.6% (5.5%) percentage weight loss. Out of 40 patients, 28 (70%) and eight (20%) patients achieved greater than 5% (5.6 kg) and 10% (11.2 kg) weight loss, respectively, as illustrated in Figure 1.

Six-month data were available for 25 semaglutide-treated patients, showing a median (IQR) weight loss of 14.9 (9.9) kg, equivalent to a percentage weight loss of 13.3% (8.4%). The thresholds of losing 5% (5.6 kg) or more, 10% (11.2 kg) or more, and 15% (16.8 kg) or more of the baseline body weight were reached in 88% (22 participants), 68% (17 participants), and 32% (eight participants), respectively. Two patients (8%) achieved more than 20% (22.4 kg) loss of the baseline body weight.

**Figure 2:**
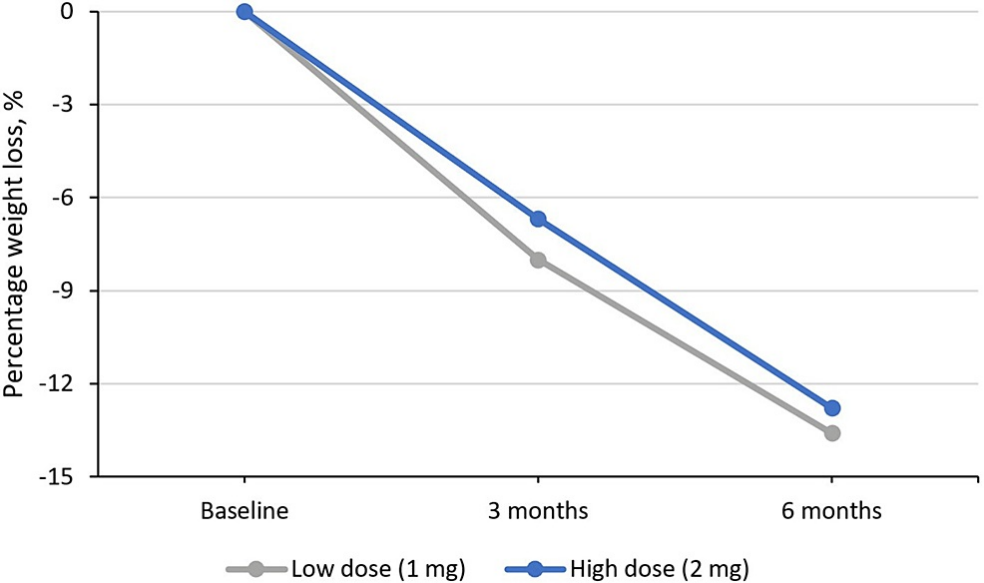
Percentage weight loss at three and six months for low and high semaglutide doses for the 25 individuals with six-month data

Three months following semaglutide initiation, the administered dose was 1 mg for all participants. Among 25 individuals who completed six-month semaglutide treatment, 16 patients received 1 mg, and the remaining nine individuals received 2 mg semaglutide dose. Individuals on 1 mg dose experienced a median weight loss of 13.6% (14.9 kg) compared to 12.8% (14 kg) in those on 2 mg dose, with weight loss greater than 10% being achieved in 10 out of 16 participants (62.5%) on 1 mg dose versus seven out of nine (77.8%) among those on 2 mg dose, as shown in Figure 2.

The total response rate was 72.5% (29 out of 40 individuals), using a cutoff of achieving a minimum 5% weight reduction within a three-month period to define clinically relevant weight loss.

Predictors of response

With regard to the effect of gender on semaglutide-induced weight loss, there was no statistically significant difference in the three-month weight change between females (median body weight reduction of 7.4 kg or 6.9%) and males (median body weight reduction of 8.2 kg or 6.4%) (P value = 0.46). However, the distribution of nine non-responders according to gender was five (out of 12 in total) males and four (out of 28) females, indicating a numerically higher percentage of 41.6% among males compared to 14.2% among females, which did not reach statistical significance (P value = 0.057). Baseline BMI was not associated with the three-month percentage weight loss (P value = 0.086). In addition, this study could not assess the impact of maintenance semaglutide dose on patient response since the decision for the titration of semaglutide dose greater than 1 mg was influenced by the magnitude of three-month weight loss, introducing a selection bias.

Patients with psychiatric disorders were overrepresented in the non-responders group, with four out of nine patients (44.4%) not showing favorable responses to semaglutide therapy. Of note, most patients (six out of nine) had depression, two anxiety, and one bipolar disorder, with the use of psychotropic medications, mostly selective serotonin reuptake inhibitors, being recorded at the time of study enrollment in seven individuals. Noteworthy, three individuals, all females with active major depressive disorder, increased their body weight during the period of semaglutide treatment. In all three cases, the period of semaglutide administration coincided with significant mental health deterioration, while none of the three patients were treated with antipsychotic medications. Regarding the effectiveness of semaglutide therapy following bariatric surgery, one individual who had undergone sleeve gastrectomy in 2008 was included, with three-month and six-month weight loss of 5.5 kg (4.9%) and 8 kg (7.1%), respectively, with mild gastrointestinal side effects. Three semaglutide-treated patients had a gastric band in situ, inserted between 2007 and 2009, showing a three-month weight loss of 2.7 kg (2.4%), 6 kg (5.6%), and 7 kg (6.0%) and experiencing in two cases mild and in one case moderate side effects. With respect to the impact of semaglutide on glycemic control, three-month semaglutide treatment resulted in the median reduction of fasting glucose by 6 mg/dL from a baseline value of 99 mg/dL, while four out of 11 participants (36.3%) with HbA1c in the range of prediabetes at the baseline reverted to normal HbA1c levels.

Adverse events

Out of 40 participants, 26 individuals (65%) reported adverse events, including 55% experiencing gastrointestinal side effects, with the most frequent being nausea, constipation, abdominal pain, diarrhea, and dyspeptic symptoms, as illustrated in Table 2. In most cases, gastrointestinal events were mild to moderate and had a short duration of less than four weeks. Severe adverse events were noted only in three patients (7.5%), with vomiting resolved in two cases within four weeks. The semaglutide discontinuation rate due to adverse events was 2.5%, with only one patient discontinuing semaglutide after seven weeks due to persistent vomiting and acute abdominal pain. The patient fully recovered, while acute pancreatitis and gallbladder-related diseases were excluded. In total, no cases of cholelithiasis, cholecystitis, or acute pancreatitis were reported.

**Table 2:** Adverse events Adverse events were classified by severity as mild (causing minimal discomfort and not interfering with everyday activities), moderate (causing sufficient discomfort to interfere with normal everyday activities), and severe (preventing normal everyday activities)

Type of adverse events	Frequency, number (%)
Any	26 (65%)
Gastrointestinal	22 (55%)
Nausea	10 (25%)
Constipation	8 (20%)
Abdominal pain	7 (17.5%)
Diarrhea	5 (12.5%)
Acid reflux	4 (10%)
Vomiting	3 (7.5%)
Halitosis	2 (5%)
Abdominal distention	2 (5%)
Flatulence	1 (2.5%)
Fatigue	1 (2.5%)
Somnolence	1 (2.5%)
Dizziness	1 (2.5%)
Cramps	1 (2.5%)
Sweats	1 (2.5%)
Severity of adverse events	Frequency, number (%)
Mild	16 (40%)
Moderate	7 (17.5%)
Severe	3 (7.5%)

## Discussion

This real-world retrospective study of semaglutide therapy for weight loss in adults without DM demonstrated a significant median six-month weight loss of 13.3% (14.9 kg), with 68% (17 out of 25 individuals) achieving at least 10% weight loss, in combination with a favorable safety profile. In almost two-thirds of the participants, substantial weight loss was produced despite being treated with 1 mg weekly, lower than the maintenance-licensed semaglutide dose of 2.4 mg weekly.

This study reported semaglutide-related percentage weight loss of 6.6% (7.4 kg) and 13.3% (14.9 kg) in three and six months, in line with the mean 6.3% and 11.8% in three and six months, respectively, observed in a similar real-world cohort study in the United States [13]. These weight loss outcomes are comparable with a three-month percentage weight loss of 6%-6.5% and six-month of 10.6%-12% reported in STEP 1 and STEP 4 trials [7,8], suggesting that semaglutide achieves a similar effect in routine clinical practice with that observed in RCTs. Therefore, an efficacy-effectiveness gap does not exist with respect to semaglutide in weight management, supporting the generalizability of RCT findings [13].

A semaglutide dose of 1 mg, lower than the approved maintenance dose of 2.4 mg per week, was prescribed in the long term for more than half the individuals in this study, leading to a median six-month weight loss of 13.6%. Another real-world study has demonstrated clinically relevant weight loss induced by low semaglutide maintenance doses, reporting a mean six-month weight loss of 9.2% in individuals receiving long-term semaglutide doses of ≤1 mg compared to 12.1% in those receiving higher doses [13]. These findings are supported by the results of a phase 2 dose-ranging trial of semaglutide in obesity, which reported a weight loss of 11.6% and 13.8% for 0.2 mg and 0.4 mg semaglutide daily dose (equivalent to 1 mg and 2.8 mg weekly dose) [17]. The role of the chronic use of lower maintenance semaglutide doses in clinical practice, especially in individuals with great initial response or persistent gastrointestinal side effects, remains unclear and needs to be explored further.

This study showed the marked variability of response to semaglutide, confirming the observation in STEP trials [7-9] that a proportion of individuals do not achieve significant weight loss for unknown reasons [11]. In our cohort, there was a trend toward higher rates of non-responders, defined as not achieving the weight loss benchmarks of either 3% after three months or 5% after six months, among males compared to females. Our observation provides evidence in favor of gender-specific differences in semaglutide-related outcomes, as already suggested by a subgroup analysis of STEP trials recording a greater mean weight reduction in females of 14%-16.2% compared to 8%-9.3% in males [18]. This study did not establish a link between starting body weight and semaglutide-related weight change, but the role of baseline BMI as a potential predictor of semaglutide response warrants further exploration in view of the clear exposure-response relationship between liraglutide and body weight reduction and the concomitant inverse relationship of baseline BMI with liraglutide-related weight loss [19]. A novel finding of this study was the overrepresentation of patients with active psychiatric disorders among non-responders. The observation in these individuals was the co-occurrence of semaglutide administration and mental health deterioration, while most of those were not on psychotropic medications related to significant weight gain. The efficacy of semaglutide in patients with active psychiatric diseases is not known since all individuals with major depressive disorders or other severe psychiatric disorders within the last two years were excluded from STEP trials [20]. In light of the bidirectional link between psychiatric disease and obesity, the weight outcomes of semaglutide in this context need to be studied [20]. In total, identifying predictors for the effectiveness of semaglutide is essential in order to optimize therapeutic benefits in the management of obesity.

This real-world study showed an early improvement in glycemic markers, confirming the glucose-lowering effect reported in STEP studies [7-9]. In addition, semaglutide improves numerous cardiovascular risk factors, including the reduction of blood pressure, total and LDL cholesterol, and triglycerides [7-9,21] and a significant decrease in C-reactive protein (CRP) concentration. In November 2023, the long-awaited results of the Semaglutide Effects on Heart Disease and Stroke in Patients with Overweight or Obesity (SELECT) trial were published, showing that semaglutide results in the 20% reduction of a composite cardiovascular end point, including death from cardiovascular causes, non-fatal myocardial infarction, and non-fatal stroke, in patients who are overweight or obese and with preexisting cardiovascular disease but without DM [22]. It remains unclear to what extent weight loss, the improvement of metabolic risk factors, and possible pleiotropic anti-atheroslcerotic effects contribute to the semaglutide-related reduction of cardiovascular risk [23].

In this real-life study, the rate of gastrointestinal side effects was 55%, mostly mild to moderate with only 7.5% of individuals experiencing serious adverse events, compared to a 74%-84% rate for gastrointestinal side effects and 7.9%-9.8% for serious side effects in STEP trials [7,9,10]. The discontinuation rate in this study was low at 2.5%, similar to the 2.9% reported in another real-world study [13] and lower than 5.9%-7.7% in STEP trials [7-9], but this difference might be explained by the longer follow-up period in RCTs. No cases of pancreatitis or gallbladder-related disease were recorded in this cohort in line with another real-world study [13], while STEP trials reported an increased likelihood for cholelithiasis at a rate of 0.8%-3.5% [7-10,12].

In contrast to the tortuous history of AOMs littered with numerous drug withdrawals due to adverse events, the mounting evidence for the good safety profile of semaglutide and its much greater magnitude of weight loss could change dramatically the landscape of obesity management, encompassing the widespread use of pharmacotherapy [24]. Following the advent of semaglutide as a potent AOM, new guidelines for the treatment of adults with obesity and weight-related complications have included a strong recommendation for using pharmacotherapy as an add-on to lifestyle interventions, when the response is inadequate, and also prioritized the use of semaglutide over other AOMs on the basis of greater weight loss [25]. However, state insurance coverage in most countries has excluded the use of AOMs, while international health technology assessment agencies have made conflicting recommendations about the reimbursement of AOMs based on their long-term cost-effectiveness [26]. The issue of high cost and limited access to AOMs becomes even more important, taking into account that obesity disproportionately affects individuals of low socioeconomic status and ethnic minorities [26]. In view of the substantial budgetary effects, negotiating the price of new-generation AOMs may be needed to address health inequities across different categories of socioeconomic status, as well as ensure their affordability and equitable patient access.

Due to its substantial and durable weight loss effect, semaglutide may herald a new era in the management of obesity, using percentage weight loss as a biomarker and applying a treat-to-target approach, in line with other chronic diseases, to prevent and treat weight-related complications [5]. Additionally, recent high-quality evidence about the cardiovascular benefit of semaglutide [22,23], indicating for the first time that the long-term use of an AOM reduces the rate of cardiovascular events, may lead to a paradigm shift, characterized by the widespread chronic administration of AOMs in individuals with obesity based not only on durable weight loss but also on cardiovascular benefit. In the future, the availability of numerous potent AOMs could usher in an era of precision medicine with the application of personalized treatment strategies, based on predictive models, encompassing age, gender, different obesity phenotypes, coexisting complications, genotype, and predictors of response to each treatment modality [27].

The main strength of this observational study is that it is the first real-life study evaluating the effectiveness and safety of semaglutide for weight management in Europe. This study has included data about a wide range of metabolic parameters and the presence of comorbidities, while all weight measurements were undertaken on the same calibrated scale, minimizing any bias potentially arising from patient-reported weight or using different scales. Nevertheless, this study has several limitations, with the first one being the absence of a comparator arm due to its observational nature, not allowing us to quantify the extent to which semaglutide and lifestyle intervention contributed to weight loss. Moreover, the lack of a strictly controlled lifestyle intervention in combination with possible differences in individual adherence introduces a potential confounder, since varying responses may be explained by different types and intensities of lifestyle modification in subgroups, rather than different drug effects per se. The second limitation is the short treatment duration, which could address neither the durability of weight loss or other metabolic effects nor the long-term safety. Third, this study included a small number of patients, not allowing subgroup analysis in order to determine differences in semaglutide response according to individual demographic characteristics and metabolic variables. Fourth, the exclusion of individuals without three-month data and the significant rate of patients discontinuing semaglutide after the first three months may introduce a selection bias, potentially overestimating its impact on body weight. Fifth, the homogenous ethnic background with all the participants being white Greek, the preponderance of females, and the bias regarding the socioeconomic status because of patients self-funding the cost of medication limit the generalizability of these findings to broader populations. Finally, a direct comparison of the weight loss effect between 1 mg and 2 mg maintenance doses was not possible since the titration decision was at the discretion of the treating clinicians, taking into account individual responses to lower doses and drug affordability.

Further research is needed to address several aspects of chronic semaglutide use for weight management, such as the durability of semaglutide-induced weight loss beyond two years [11], the optimal duration of treatment, and strategies to prevent or, at least, ameliorate weight regain occurring after semaglutide discontinuation [8,28]. Marked variability in the therapeutic result of semaglutide highlights the need to identify the predictors of response and assess the efficacy of lower semaglutide doses, facilitating personalized decision-making. Future research efforts should not only evaluate the potential synergistic effect of combining semaglutide with other AOMs and different lifestyle interventions but also explore its use as neoadjuvant or adjuvant therapy along with bariatric surgery. In addition, a new generation of studies should recognize distinct obesity phenotypes and evaluate the use of semaglutide for weight management in patient subgroups with different main comorbidities with primary end points other than weight, for example, the progression of liver steatohepatitis or the regression of liver fibrosis [29]. In view of the potential widespread use of semaglutide, cost-effectiveness studies are of paramount importance, as well as head-to-head studies with other AOMs. Despite the favorable risk profile of semaglutide in STEP trials, postmarketing surveillance with the employment of prospective databases should assess whether semaglutide is associated with an increased risk for gastroparesis and gastrointestinal ileus, as suggested by a recent study [30], or alters the risk for rare complications with a long latency period, such as the incidence of malignancies. Finally, concerns about the possible association of semaglutide-induced weight loss with sarcopenia and the loss of bone density should be addressed, and if needed, strategies should be developed to ameliorate these unwanted effects.

## Conclusions

This study showed the great effectiveness of semaglutide in a real-life setting, leading to a median three-month and six-month weight loss of 6.6% (7.4 kg) and 13.3% (14.9 kg), respectively, with 68% (17 out of 25 individuals) attaining 10% weight loss over six months. Of note, a substantial proportion of individuals, administered with a lower-than-approved maintenance dose of 1 mg, achieved significant weight loss. Semaglutide was well tolerated, with adverse gastrointestinal events being mostly mild to moderate and brief in duration, leading to discontinuation in only one case (2.5%). These findings suggest that the results of STEP trials can be extrapolated and generalized to routine clinical practice. Future studies with a longer follow-up are warranted to confirm the real-life effectiveness and safety of semaglutide for weight management, to examine the potency of lower-than-approved maintenance doses, and to identify the predictors of response, facilitating a personalized therapeutic approach. Breaking barriers, such as high cost, the lack of reimbursement, and medication shortages, is essential to ensure affordability and equitable access to effective AOMs. Therefore, the advent of semaglutide could represent a paradigm shift in obesity care, encompassing the widespread long-term use of pharmacotherapy across lifestyle interventions and employing a personalized treat-to-target approach, similar to the contemporary management of type 2 DM, in order to prevent and treat weight-related complications.
